# Bloodstream infections and local access site infection surveillance program in hemodialysis, Vaud, Switzerland

**DOI:** 10.1186/2047-2994-4-S1-P63

**Published:** 2015-06-16

**Authors:** A Deschamps, M Attinger, D Zbinden, C Petignat

**Affiliations:** 1 Unité Cantonale HPCI Vaudoise, Lausanne, Switzerland

## Introduction

Patients on chronic hemodialysis (HD) are at high risk of developing bloodstream infections (BSI) and local access site infection (LASI). These infections engender high morbidity and mortality. In 2007, a BSI and LASI infections surveillance program was created, following the implementation of recommendations for the prevention of healthcare-associated infections in HD centers of the canton of Vaud. Between 2007-2013, this surveillance program encountered organizational difficulties and failed to produce usable data representative of the situation.

## Objectives

The aim of the intervention was to improve the structure of the surveillance program in the different HD centers of the canton of Vaud to produce usable data representative of the situation.

## Methods

In order to improve infection data collection from the HD centers, a nurse was designated as Surveillance nurse in each center. The questionnaire and work instructions created in 2007 were adapted. A standardized methodological framework was established. Surveillance nurses in the participating centers were audited on work methodology. They were trained by the Senior Infection Control Nurse Specialist to better recognize and report infections in the questionnaire and they were offered regular contact to discuss problems encountered. The Senior Infection Control Nurse Specialist received the questionnaires and entered data to be analyzed on a database.

## Results

The number of reported infections is increasing in particular regarding LASI. 54 episodes of BSI and LASI were reported in 2014 compared to 16 reported episodes in 2013, for an equivalent number of HD sessions. In 2013, among the 16 episodes, 12 were BSI and 4 were LASI (3 catheter infections and 1 fistula infection). In 2014, 30 were BSI and 23 were LASI (21 catheter infections and 2 fistula infections).

## Conclusion

Auditing and training nurses in charge in HD centers helped us to increase the quality of BSI and LASI reporting. We observed an increased number of reported infections, especially LASI. The LASI could not be easily detected before the implementation of the methodological framework. This surveillance improvement ensures a more precise data collection enabling us to be more accurate in describing the epidemiology of BSI and LASI.

## Disclosure of interest

None declared.

